# Dyslipidemia among rural and urban HIV patients in south-east Malawi

**DOI:** 10.1371/journal.pone.0197728

**Published:** 2018-05-21

**Authors:** Alemayehu Amberbir, Victor Singano, Alfred Matengeni, Zahra Ismail, Gift Kawalazira, Adrienne K. Chan, Sumeet D. Sodhi, Joep J. van Oosterhout

**Affiliations:** 1 Dignitas International, Zomba, Malawi; 2 Pirimiti Rural Hospital, Pirimiti, Malawi; 3 District Health Office, Ministry of Health, Zomba, Malawi; 4 Division of Infectious Diseases, Department of Medicine, Sunnybrook Health Sciences Centre, University of Toronto, Toronto, Canada; 5 Toronto Western Hospital, University Health Network, Toronto, Canada; 6 Department of Medicine, College of Medicine, University of Malawi, Blantyre, Malawi; University of Liverpool, UNITED KINGDOM

## Abstract

**Background:**

While dyslipidemia importantly contributes to increased cardiovascular disease risk among patients on antiretroviral therapy (ART), data on lipid patterns among African adults on ART are limited. We describe the prevalence of lipid abnormalities and associated factors in two HIV clinics in Malawi.

**Methods:**

We conducted a cross-sectional study in 2014 and enrolled adult patients at a rural and an urban HIV clinic in Zomba district, Malawi. We recorded patient characteristics, CVD risk factors and anthropometric measurements, using the WHO STEPS validated instrument. Non-fasting samples were taken for determination of total cholesterol (TC), triglyceride (TG) and HDL-cholesterol (HDL-c) levels. Logistic regression analysis was used to determine factors associated with elevated TC and elevated TC/HDL-c ratio.

**Results:**

554 patients were enrolled, 50% at the rural HIV clinic, 72.7% were female, the median (IQR) age was 42 years (36–50); 97.3% were on ART, 84.4% on tenofovir/lamivudine/efavirenz, 17.5% were overweight/obese and 27.8%% had elevated waist/hip ratio. 15.5% had elevated TC, 15.9% reduced HDL-c, 28.7% had elevated TG and 3.8% had elevated TC/HDL-c ratio. Lipid abnormalities were similar in rural and urban patients. Women had significantly higher burden of elevated TC and TG whereas men had higher prevalence of reduced HDL-c. Waist-to-hip ratio was independently associated with elevated TC (aOR = 1.90; 95% CI: 1.17–3.10, p = 0.01) and elevated TC/HDL-c ratio (aOR = 3.50; 95% CI: 1.38–8.85, p = 0.008). Increasing age was independently associated with elevated TC level (aOR = 1.54, 95% CI 0.51–4.59 for age 31–45; aOR = 3.69, 95% CI 1.24–10.95 for age >45 years vs. ≤30 years; p-trend <0.01).

**Conclusions:**

We found a moderate burden of dyslipidemia among Malawian adults on ART, which was similar in rural and urban patients but differed significantly between men and women. High waist-hip ratio predicted elevated TC and elevated TC/HDL-c ratio and may be a practical tool for CVD risk indication in resource limited settings.

## Introduction

The wide roll-out of antiretroviral therapy (ART) for people living with HIV (PLHIV) in sub-Saharan Africa [1] has shifted the burden of HIV-related morbidity to chronic conditions including cardiovascular diseases (CVD) [2,3]. Several studies have demonstrated an increased risk of myocardial infarction and stroke in PLHIV compared with uninfected controls [2,4]. High prevalence of classical CVD risk factors such as hypertension, diabetes, smoking and dyslipidemia, as well as chronic inflammation and immune activation associated with HIV infection, contribute to the increased risk of CVD among PLHIV [5,6]. HIV infection and early ART are risk factors for stroke in Malawian adults with the highest attributable risk in younger patients [2]. Individual antiretroviral drugs have also been linked to CVD through elevated markers of atherosclerosis [7,8].

Among risk factors for CVD in PLHIV, dyslipidemia has received limited attention in sub-Saharan Africa. A systematic review and meta-analysis of data from the region found that HIV infection was associated with higher triglyceride (TG) and lower HDL-cholesterol (HDL-c) levels and ART was associated with increased LDL-cholesterol levels [6]. In Ethiopia, patients on ART had higher levels of total cholesterol (TC) and TGs, and lower HDL-c compared to ART-naïve subjects [7]. Studies from the region mainly included patients from urban areas, where a less physically active life style and unhealthy diet may have an unfavorable impact on lipid patterns. Furthermore, earlier studies often enrolled patients on stavudine based regimens, leaving them more prone to the metabolic syndrome, lipodystrophy and dyslipidemia [9].

Patterns of dyslipidemia, its risk factors and the consequences for CVD events among African patients on current ART regimens need to be better understood to determine implications for lipid monitoring in high HIV prevalence and low-income countries. We have therefore described the prevalence of dyslipidemia and associations with patient characteristics in a rural and an urban HIV clinic in south-east Malawi.

## Methods

### Study design and setting

This is an analysis of a larger cross-sectional study that we conducted between July and October 2014. We enrolled consecutive HIV infected adults (aged 18 years or older) in care at the HIV clinics of Pirimiti Rural Hospital and Zomba Central Hospital (urban). The first 277 participants at each clinic received a lipid measurement and were included in the current analysis. The clinics are located in Zomba district, southern Malawi, where HIV prevalence is around 14.5% in the 15–49 year age group [10]. During the study period, the urban clinic served an estimated 6,700 HIV infected patients and the rural clinic 3,200. Further details of the study enrolment procedures and methods were reported elsewhere [5]. The study was approved by the Malawi College of Medicine Research and Ethics Commission (approval number P.02/14/1523). All study participants provided their informed written consent.

### Data collection and measurements

We used adapted, validated WHO STEPS questionnaires to collect information related to CVD risk factors [11]. Information about smoking and alcohol consumption was collected and the level of physical activity was categorized as active and less active [5]. HIV related information was extracted from standard clinic registers and ART master cards. This included ART status, type of ART regimen, ART duration and CD4 count at ART initiation. Anthropometric measurements (weight, height, waist and hip circumferences) were taken by a trained research nurse following the WHO STEPS guide to physical measurements procedure. Body mass index (BMI) was categorized as normal, underweight, overweight and obese using the WHO classification [12]. Central obesity was defined as waist-hip- ratio (waist circumference/hip circumference) of ≥0.90 for men and ≥0.85 for women [13]. The Framingham risk score (FRS) score, which includes sex, current smoking status, total cholesterol, HDL-cholesterol, anti-hypertensive drug use and systolic blood pressure was calculated for all patients aged ≥20 years as recommended [14]. Non-fasting samples were taken for measurement of TC, TG and HDL-c levels, using the point-of-care CardioChek® PA (Polymer Technology Systems, Inc. Indianapolis, USA). TC was elevated if >5.2mmol/L, HDL-c was decreased if <1.1 mmol/L and TG was elevated if >2.9 mmol/L. The TC/HDL-c ratio was used to indicate a lipid profile prone to atherosclerosis and considered elevated if >5.0.[15]

### Statistical analysis

Laboratory and questionnaire data were entered in an Access database, cleaned and analysed using Stata 13 (Statacorp, College Station Texas, USA). Elevated TC and elevated TC/HDL-c ratio were considered as primary markers of increased cardiovascular risk. Lipid data were stratified by urban/rural study site as well as gender. Associations of lipid findings with lifestyle, metabolic and HIV related risk factors were analyzed using crude odds ratios (ORs) with 95% confidence intervals (CI). Independent predictors of elevated TC and elevated TC/HDL-c ratio were determined with logistic regression analysis adjusting for various confounders. Variables with a p-value of ≤0.1 in univariate analyses were included in the multivariable logistic regression models. Age, sex and urban/rural site were also included in the model as *a priori* confounders, irrespective of p-values in the univariable analysis. We report overall p-values and p-values for trend for ordinal variables with more than two categories.

## Results

### Description of the study participants

A total of 554 patients were enrolled in the study, 277 (50.0%) were from the rural HIV clinic, 403 (72.7%) were female, the median (IQR) age was 42 years (36–50); 539 (97.3%) were on ART (79.0% for more than 2 years) and 455 (84.4%) were on the standard first line regimen tenofovir/lamivudine/efavirenz. At ART initiation 246 patients (67.0%) had CD4 levels of ≤ 250 mm^3^ and 207 (38.3%) were in the WHO disease stage III/IV. Ninety seven (17.5%) were overweight/obese, the median (IQR) BMI was 21 (20–24)) and 169 (31.3%) had an elevated waist/hip ratio, median (IQR) (0.82 (0.79–0.88)). The majority (90.6%) reported being active (vs. less active) in terms of physical activity, 63 (11.4%) reported using alcohol in the past 12 months and only 18 (3.3%) said they were current smokers. The Framingham risk score (FRS) could be determined in 541 patients and the 10-year CVD event risk was ≥10% in 29 patients (5.4%).

### Prevalence of dyslipidemia by rural/urban site and gender

Eighty-six patients (15.5%) had elevated TC, 88 (15.9%) had reduced HDL-c, 159 (28.7%) had elevated TGs, 21 (3.8%) had elevated TC/HDL-c ratio and 283 (51.1%) had completely normal lipid profiles. There were no significant differences in dyslipidemia between rural and urban patients (Table 1). Women had a significantly higher burden of elevated TC (17.6% vs. 9.9%; p = 0.026) and elevated TGs (32.3% vs. 19.2%; p = 0.002) whereas men had higher prevalence of reduced HDL-c (25.2% vs. 12.4%; p<0.01) (Table 1). Patients with elevated TC levels and with elevated TC/ HDL-c ratio were more likely to have a FRS 10-year CVD risk ≥10% than those with normal TC levels (13.3% vs. 3.9%; p = 0.001) and a normal HDL-c/TC ratio (23.8% vs. 4.6%; p<0.001) respectively.

**Table 1:** Lipid levels among Malawian, adult HIV patients stratified by urban/rural site and by gender.

Lipid profiles *(N = 554)*	Total N (%)	Rural n (%)	Urban n (%)	P-value*	Female n (%)	Male n (%)	P-value*
*Total cholesterol*				0.814			**0.026**
Elevated	86 (15.5)	44 (15.9)	42 (15.2)		71 (17.6)	15 (9.9)	
Normal	468 (84.5)	233 (84.1)	235 (84.8)		332 (82.4)	136 (90.1)	
*Mean total cholesterol (SD)*	4.14 (1.02)	4.18 (0.99)	4.10 (1.04)	0.173	4.23 (1.05)	3.89 (0.88)	**<0.01**
*HDL cholesterol*				0.642			**<0.01**
Low	88 (15.9)	46 (16.6)	42 (15.2)		50 (12.4)	38 (25.2)	
Normal	466 (84.1)	231 (83.4)	235 (84.8)		353 (87.6)	113 (74.8)	
*Mean HDL cholesterol (SD)*	1.60 (0.82)	1.58 (0.74)	1.62 (0.89)	0.682	1.65 (0.84)	1.46 (0.73)	**0.006**
*Total/HDL cholesterol ratio*				0.824			0.890
Elevated	21 (3.8)	11 (4.0)	10 (3.6)		15 (3.7)	6 (4.0)	
Normal	533 (96.2)	266 (96.0)	267 (96.4)		388 (96.3)	145 (96.0)	
*Mean total/HDL cholesterol (SD)*	2.91 (1.04)	2.95 (1.04)	2.87 (1.04)	0.177	2.86 (1.02)	3.02 (1.10)	0.946
*Triglycerides*				0.159			**0.002**
Elevated	159 (28.7)	87 (31.4)	72 (26.0)		130 (32.3)	29 (19.2)	
Normal	395 (71.3)	190 (68.6)	205 (74.0)		273 (67.7)	122 (80.8)	
*Mean triglycerides (SD)*	2.51 (3.57)	2.72 (4.87)	2.31 (1.33)	0.088	2.69 (4.11)	2.03 (1.16)	0.026*

SD, standard deviation; HDL, high density lipoprotein cholesterol

* P-value based on result of two-way sample t-test with equal variances or chi-square test, as applicable

### Factors associated with elevated TC/HDL-c ratio and TC

In multivariable logistic analysis adjusted for various confounders, waist-to-hip ratio (aOR = 1.90; 95% CI: 1.17–3.10, p = 0.01), and increasing age (age 31–45: aOR = 1.54, 95% CI 0.51–4.59; age >45 years: aOR = 3.69, 95% CI 1.24–10.95 vs. ≤30 years; p-trend <0.001) were independently associated with elevated TC level (Table 2). Only waist-to-hip ratio (aOR = 3.50; 95% CI: 1.38–8.85, p = 0.008) was independently associated with elevated TC/HDL-c ratio (Table 3). There was no significant association between elevated TC/HDL-c ratio or TC and urban/rural study site, gender, diagnosis of diabetes, body mass index, ART status, duration of ART, CD4 levels at ART initiation, levels of physical activity and current use of alcohol (Tables 2 and 3).

**Table 2:** Multivariate analysis–factors associated with elevated total cholesterol (TC) in Malawi.

*Characteristics*	*Total* *n/N (%)*	*Elevated TC n (%)*	*Crude Odds* *Ratio (95% CI)*	*P-value*	*Adjusted Odds* *Ratio (95% CI)*	*P-value*
*Study site (N = 554)*				0.814		0.696
Urban	277 (50.0)	42 (15.2)	0.95 (0.60, 1.50)		0.91 (0.56, 1.47)	
Rural	277 (50.0)	44 (15.9)	1		1	
*Age in years (N = 554)*				<0.001* <0.001 ^**¥**^		<0.001* <0.001^**¥**^
≤30	51 (9.2)	4 (7.8)	**1**		**1**	
31–45	302 (54.5)	35 (11.6)	**1.54 (0.52, 4.54)**		**1.54 (0.51, 4.59)**	
>45	201 (36.3)	47 (23.8)	**3.59 (1.23, 10.47)**		**3.69 (1.24, 10.95)**	
*Sex (N = 554)*				**0.028**		0.064
Male	151 (27.3)	15 (9.9)	**0.52 (0.29, 0.93)**		0.54 (0.28, 1.04)	
Female	403 (72.7)	71 (17.6)	**1**		1	
*ART Status (N = 554)*				-		
On ART	539 (97.3)	86 (16.0)	-			
Pre-ART	15 (2.7)	0 (0.0)	-			
*ART regimen (N = 539)*				0.501*		
TDF/3TC/EFV	455 (84.4)	69 (15.2)	0.83 (0.23, 2.98)			
AZT/3TC/NVP	67 (12.4)	14 (20.9)	1.23 (0.31, 4.90)			
Other	17 (3.2)	3 (17.7)	1			
*ART duration in months (N = 539)*				0.138		
<24	114 (21.2)	13 (11.4)	1			
≥24	425 (78.9)	73 (17.2)	1.61 (0.86, 3.03)			
*CD4 count at ART initiation (N = 367)*				0.117*		
≤250	246 (67.0)	46 (18.7)	1			
>250	121 (32.3)	13 (10.7)	0.52 (0.27, 1.01)			
Missing	187 (33.8)	27 (14.4)	0.73 (0.44, 1.23)			
*Diagnosis of diabetes (N = 554)*				0.230		
No	524 (94.6)	79 (15.1)	1			
Yes	30 (5.4)	7 (23.3)	1.71 (0.71, 4.13)			
*Body mass index (Kg/m*^*2*^*) (N = 554)*				0.462* 0.338^¥^		
Normal	389 (70.2)	58 (14.9)	1			
Underweight	68 (12.3)	9 (13.2)	0.87 (0.41, 1.85)			
Overweight/obesity	97 (17.5)	19 (19.6)	1.39 (0.78, 2.47)			
*Waist–to-hip ratio (N = 540)*				**0.003**		**0.01**
Normal	371 (68.7)	46 (12.4)	**1**		**1**	
High	169 (31.3)	38 (22.5)	**2.05 (1.27, 3.30)**		**1.90 (1.17, 3.10)**	
*Physical activity (N = 554)*				0.118		
Less active	52 (9.4)	12 (23.1)	1			
Active	502 (90.6)	74 (14.7)	0.58 (0.29, 1.15)			
*Alcohol use in the past 12 months (N = 554)*				**0.041**		0.133
No	491 (88.6)	82 (16.7)	**1**		1	
Yes	63 (11.4)	4 (6.4)	**0.34 (0.12, 0.96)**		0.44 (0.14, 1.29)	
*Current smokers (N = 554)*				0.261		
No	536 (96.8)	85 (15.9)	1			
Yes	18 (3.3)	1 (5.6)	0.31 (0.04, 2.38)			

TDF, tenofovir; 3TC, lamivudine; EFV, efavirenz; AZT, zidovudine; NVP, nevirapine

*P-value based on result of LHR test

^¥^P-value for trend; age, sex and study site were included in the model as *a priori* confounder regardless of degree of association.

**Table 3:** Multivariate analysis–factors associated with elevated TC/HDL-c ratio in Malawi.

*Characteristics*	*Total* *n/N (%)*	*Elevated TC/HDL-c ratio n (%)*	*Crude Odds* *Ratio (95% CI)*	*P-value*	*Adjusted Odds* *Ratio (95% CI)*	*P-value*
*Study site (N = 554)*				0.824		0.996
Urban	277 (50.0)	10 (3.6)	0.91 (0.38, 2.17)		1.00 (0.40, 2.48)	
Rural	277 (50.0)	11 (4.0)	1		1	
*Age in years (N = 554)*				0.736* 0.636^¥^		0.804* 0.645^¥^
≤30	51 (9.2)	1 (2.0)	1		1	
31–45	302 (54.5)	12 (4.0)	2.07 (0.26, 16.26)		1.84 (0.23, 14.85)	
>45	201 (36.3)	8 (4.0)	2.07 (0.25, 16.96)		1.92 (0.23, 16.09)	
*Sex (N = 554)*				0.890		0.799
Male	151 (27.3)	6 (4.0)	1.07 (0.41, 2.81)		1.15 (0.40, 3.31)	
Female	403 (72.7)	15 (3.7)	1		1	
*ART Status (N = 554)*				-		
On ART	539 (97.3)	21 (3.9)	-			
Pre-ART	15 (2.7)	0 (0.0)	-			
*ART regimen (N = 539)*				0.780*		
TDF/3TC/EFV	455 (84.4)	20 (4.4)	0.74 (0.09, 5.83)			
AZT/3TC/NVP	67 (12.4)	0 (0.0)	-			
Other	17 (3.2)	1 (5.9)	1			
*ART duration in months (N = 539)*				0.810		
<24	114 (21.2)	4 (3.5)	1			
≥24	425 (78.9)	17 (4.0)	1.15 (0.38, 3.47)			
*CD4 count at ART initiation (N = 367)*				0.080*		
≤250	246 (67.0)	12 (4.9)	1			
>250	121 (32.3)	1 (0.8)	0.16 (0.02, 1.26)			
Missing	187 (33.8)	8 (4.3)	0.87 (0.35, 2.18)			
*Diagnosis of diabetes (N = 554)*				0.893		
No	524 (94.6)	20 (3.8)	1			
Yes	30 (5.4)	1 (3.3)	0.87 (0.11, 6.70)			
*Body mass index (Kg/m*^*2*^*) (N = 554)*				0.202* 0.088^¥^		
Normal	389 (70.2)	12 (3.1)	1			
Underweight	68 (12.3)	2 (2.9)	0.95 (0.21, 4.35)			
Overweight/obesity	97 (17.5)	7 (7.2)	2.44 (0.94, 6.38)			
*Waist–to-hip ratio (N = 540)*				**0.008**		**0.008**
Normal	371 (68.7)	8 (2.2)	**1**		**1**	
High	169 (31.0)	12 (7.1)	**3.47 (1.39, 8.65)**		**3.50 (1.38, 8.85)**	
*Physical activity (N = 554)*				0.133		
Less active	52 (9.4)	4 (7.7)	1			
Active	502 (90.6)	17 (3.4)	0.42 (0.14, 1.30)			
*Alcohol use in the past 12 months (N = 554)*				0.786		
No	491 (88.6)	19 (3.9)	1			
Yes	63 (11.4)	2 (3.2)	0.81 (0.19, 3.58)			
*Current smokers (N = 554)*				-		
No	536 (96.8)	21 (3.9)	-			
Yes	18 (3.3)	0 (0.0)	-			

TDF, tenofovir; 3TC, lamivudine; EFV, efavirenz; AZT, zidovudine; NVP, nevirapine

*LHR test

^¥^P-value for trend; age, sex and study site were included in the model as *a priori* confounder regardless of degree of association.

## Discussion

In this study of Malawian adults in HIV care, we documented a moderate burden of dyslipidemia. Nearly all (97.3%) were on ART, and a large majority was on a regimen that is the current standard in the region. Most patients (72.7%) were female and the proportion of elevated waist-hip ratio was substantial (27.8%). Half of the patients had a normal lipid spectrum. The burden of abnormal lipid levels was similar in urban and rural patients but differed significantly between men and women. The prevalence of FRS elevated CVD risk, i.e. the probability of a CVD event in the next decade being >10%, was quite low (5.4%) overall but in those with elevated TC and TC/HDL-c ratio it was considerable (13.3% and 23.8% respectively). Elevated waist-to-hip ratio, but not high BMI, was independently associated with elevated TC level and elevated TC/HDL-c ratio.

A systematic review and meta-analysis of cardio-metabolic risk factors, HIV and ART in sub-Saharan Africa included 52 studies from 14 counties of nearly 30,000 individuals found that among PLHIV those on ART had higher LDL-c and HDL-c levels, TG level was not significantly different, while TC was not reported [6]. Many studies in this review were from an era when stavudine and nevirapine containing regimens were used, whereas in our study and in most HIV programmes in sub-Saharan Africa, these drugs have been replaced with tenofovir and efavirenz. Stavudine has particularly unfavorable effects on TC, TG and LDL-c levels [16,17]. Nevirapine may have some benefit over efavirenz in terms of increasing HDL-c and being associated with better surrogate markers of atherosclerosis [17]^.^ This difference with nevirapine may be attributed to a subgroup of efavirenz users with high drug levels, as higher efavirenz levels were associated with moderate increases in TC, TG, HDL-c and LDL-c in a study of 106 South Africans on efavirenz-based ART [18].

Because the composition of standardized first-line ART regimens is relevant to lipid levels, it is important to compare our study with more recent studies from the region. In a study from urban Tanzania that enrolled 354 patients on ART, the burden of dyslipidemia was considerably higher than in our study (elevated TC 71.6%, elevated TG 67.0%, low HDL-c 43.6%), but various, not clearly specified ART regimens were used, more patients were obese and the prevalence of central obesity was also higher [19]. Two hundred fifty Ugandan adults who were on ART for at least two years were enrolled in a cross sectional study of CVD risk factors [20]. Participants had similar gender and age characteristics as ours and comparable median duration on ART. Fifty percent used tenofovir/lamivudine/efavirenz, none were on stavudine. Prevalence of elevated TC was 6.4%, elevated TG 29.6%, low HDL-c 85.6% and elevated TC/HDL-c ratio 22.4%. Another cross sectional study from Uganda investigated 1,024 adults on long-term ART (median duration 9.6 years) but did not specify ART regimens other than protease inhibitor-based or not [21]. Similar to the other Ugandan study [20], being overweight/obese (26.1%) and having abdominal obesity (52.6%) were very common. In this study the prevalence of elevated TC was 30.2%, elevated TG 21.2%, and low HDL-c 37.5% [21]. Among 400 patients on ART from Cameroon, 79.9% were female, the median age was 44 years, 50.8% were obese, 45.5% had abdominal obesity and 93.3% were on zidovudine/lamivudine plus efavirenz or nevirapine, with a median duration of 3 years [22]. In this population, the prevalence of elevated TC was 6.3%, of elevated TG 7.8% and of low HDL-c 19.5% [22]. The large variation in dyslipidemia observed in these sub-Saharan African studies, including ours, all published in the last four years may be explained by differences in genetic factors, overweight/obesity, central obesity, ART duration, ART regimens and (unmeasured) ART failure rates between the study populations. Very few prospective studies are available and none reported results from regular lipid monitoring and dyslipidemia treatment results. While life style, including physical activity, dietary habits, consumption of alcohol and smoking may vary by geographical setting, we found that lipid abnormalities were not significantly different between rural and urban patients on similar ART regimens. We did not find other studies that compared lipid patterns between urban and rural ART patients.

Women had a 1.8 times higher prevalence of elevated TC and a 1.7 times higher prevalence of elevated TGs. In men, the prevalence of reduced HDL-c was twice as high as in women. In Ugandan ART patients the burden of abnormal TC, TG and HDL-c levels was not significantly different between men and women [20]. The prevalence of dyslipidemia (defined as elevated TC and/or low HDL-c) was also similar in males and females in a study of HIV negative and HIV positive, ART-naive adult Kenyans [23]. Among Ugandans on stable ART, mean HDL-c levels were significantly lower in men and mean TG levels significantly higher in women, but dyslipidemia was not reported by gender [21]. Mean TC and mean HDL-c levels were not significant different between men and women in a small Zimbabwean study of HIV positive adults who were mostly ART experienced [24]. Contrary to several smaller studies from the region, we may have detected gender differences in dyslipidemia due to our relatively large sample size, but our findings need confirmation in similar settings.

Our study found that waist-to-hip ratio was the only characteristic that predicted both elevated TC/HDL-c ratio and elevated TC. Elevated waist-to-hip ratio resulted in a nearly 2-fold increased probability of elevated TC and a 3.5-fold higher probability of elevated TC/HDL-c ratio. A literature review, not HIV focussed, concluded that both general and abdominal obesity are associated with CVD risk factors and incident CVD events [25]. It further showed that measures of central obesity do not have better discriminatory capacity as predictors of dyslipidaemia and CVD risk than BMI, and that they are not more suitable outside Caucasian populations. The quality of the evidence regarding the latter points was limited, in particular due to the cross sectional nature of most studies [25]. A large pooled analysis >200,000 Asians similarly found that BMI and waist-to-hip ratio were both associated with lipid parameters. The authors concluded that measures of central obesity may help to identify individuals at increased risk of dyslipidaemia [26]. We did not find studies from African HIV populations that reported associations of different measures of obesity with dyslipidaemia and individual lipid parameters. Given the feasibility of measuring central obesity in resource limited settings, our results, if confirmed, suggest that waist-hip ratio may be utilized for clinical decision making in ART clinics, for instance to select patients for lipid measurements.

Countries in Africa are experiencing a rapid epidemiologic transition, due to population ageing and lifestyle changes (urbanization, diet, tobacco and alcohol consumption and physical inactivity). Such changes together with an increasing burden of obesity could be the drivers of dyslipidemia in this population.[27] In a large population based study in Malawi; we recently found that being overweight and obese are highly prevalent in urban and rural Malawian adults, especially in the 35–54 years age range and in women, and is comparable to that observed in high-income countries.[27] HIV infection itself may play a role on dyslipidemia through endothelial cell injury and local inflammatory response that may impair vessel responsiveness.[3,28]

Our study needs to be interpreted in the light of its limitations. The cross-sectional design precludes causal associations between dyslipidemia and patient characteristics. The study did not include a control group of HIV-uninfected persons which would have provided better insight into the role of HIV infection and antiretroviral drugs. We measured lipid profiles using non-fasting samples but these do not differ significantly from results obtained from fasting samples for clinical interpretation, have similar associations with CVD risk and have recently been recommended in favor of fasting samples due to higher feasibility [29]. We used reference values from western settings that have not been validated in sub-Saharan Africans to classify dyslipidemia, as did other studies from the region. The main strength of this study is our ability to compare gender, urban vs. rural location and other patient characteristics among ART patients on current (tenofovir/lamivudine/efavirenz) regimens and that we included relevant anthropomorphic measures, in particular waist-hip-ratio.

## Conclusions

We found a moderate burden of dyslipidemia among Malawian adults on current ART regimens. Dyslipidemia was similar in patients in rural and urban settings but patterns differed significantly between men and women. Patients with elevated TC and elevated TC/HCL-c ratio had significantly higher FRS predicted CVD risk. Higher age was associated with elevated TC/HCL-c ratio. Elevated waist-to-hip ratio predicted both elevated TC and elevated TC/HCL-c ratio and may be a feasible tool for prediction of dyslipidemia and CVD risk. Future prospective studies with CVD endpoints among African ART patients are needed to determine the role of lipid monitoring and treatment of dyslipidemia.
